# Change over Time in the Risk of Death among Japanese COVID-19 Cases Caused by the Omicron Variant Depending on Prevalence of Sublineages

**DOI:** 10.3390/ijerph20042779

**Published:** 2023-02-04

**Authors:** Yuki Takahashi, Hideo Tanaka, Yoshitaka Koga, Shunichi Takiguchi, Shigeru Ogimoto, Shizuyo Inaba, Hiroyuki Matsuoka, Yuka Miyajima, Takeshi Takagi, Fujiko Irie, Yoshihito Bamba, Fuyo Yoshimi, Tomoyuki Suzuki, Isao Araki, Chika Shirai, Sayuri Matsumoto, Motoyuki Shimizu, Toshiyuki Shibata, Hitomi Nagai, Masaru Kinoshita, Rie Fujita, Tsuyoshi Ogata

**Affiliations:** 1 Fujiidera Public Health Center of Osaka Prefectural Government, Fujiidera 583-0024, Japan; 2 Neyagawa City Public Health Center, Neyagawa 572-0838, Japan; 3 Tosu Public Health and Welfare Office of Saga Prefectural Government, Tosu 841-0051, Japan; 4 Central Public Health Center of Miyazaki Prefectural Government, Kirishima 880-0032, Japan; 5 Miyoshi Public Health Center of Tokushima Prefectural Government, Mima 778-0002, Japan; 6 Gifu Public Health Center of Gifu Prefectural Government, Gifu 504-0838, Japan; 7 Iida Public Health Center of Nagano Prefectural Government, Iida 395-0034, Japan; 8 Matsumoto Public Health Center of Nagano Prefectural Government, Matsumoto 390-0852, Japan; 9 Isesaki Public Health Center of Gunma Prefectural Government, Isesaki 372-0024, Japan; 10 Tsuchiura Public Health Center of Ibaraki Prefectural Government, Tsuchiura 300-0812, Japan; 11 Ibaraki Prefectual Department of Public Health and Welfare, Mito 310-8555, Japan; 12 Central Public Health Center of Ibaraki Prefectural Government, Mito 310-0852, Japan; 13 Shiga Prefectural Department of Public Health Care and Welfare, Otsu 520-8577, Japan; 14 Shiga Prefectural Health, Medical and Welfare Department, Otsu 520-8577, Japan; 15 Hirakata City Public Health Center, Hirakata 573-0027, Japan; 16 Higashiosaka City Public Health Center, Higashiosaka 578-0941, Japan; 17 Suita City Public Health Center, Suita 564-0072, Japan; 18 Ibaraki Public Health Center of Osaka Prefectural Government, Ibaraki 567-8585, Japan; 19 Kenou Public Health Center of Nagasaki Prefectural Government, Isahaya 854-0081, Japan; 20 Itako Public Health Center of Ibaraki Prefectural Government, Itako 311-2422, Japan

**Keywords:** SARS-CoV-2, Omicron variant, case fatality rate, Japanese

## Abstract

To assess temporal changes to the risk of death in COVID-19 cases caused by the Omicron variant, we calculated age-standardized case fatality rates (CFR) in patients aged ≥40 years over nine diagnostic periods (3 January to 28 August 2022) in ten Japanese prefectures (14.8 million residents). Among 552,581 study subjects, we found that there were 1836 fatalities during the isolation period (up to 28 days from date of onset). The highest age-standardized CFR (0.85%, 95% confidence interval (CI):0.78–0.92) was observed in cases diagnosed in the second 4-week period (January 31 to February 27), after which it declined significantly up to the 6th 4-week period (0.23%, 95% CI: 0.13–0.33, May 23 to June 19). The CFR then increased again but remained at 0.39% in the eighth period (July 18 to August 28). The CFR in cases with the BA.2 or BA.5 sublineages in the age range 60–80 years was significantly lower than that with BA.1 infections (60 years: 0.19%, 0.02%, 0.053%, respectively; 70 years: 0.91%, 0.33%, 0.39%; ≥80 years: 3.78%, 1.96%, 1.81%, respectively). We conclude that the risk of death in Japanese COVID-19 patients infected with Omicron variants declined through February to mid-June 2022.

## 1. Introduction

**Figure A1:**
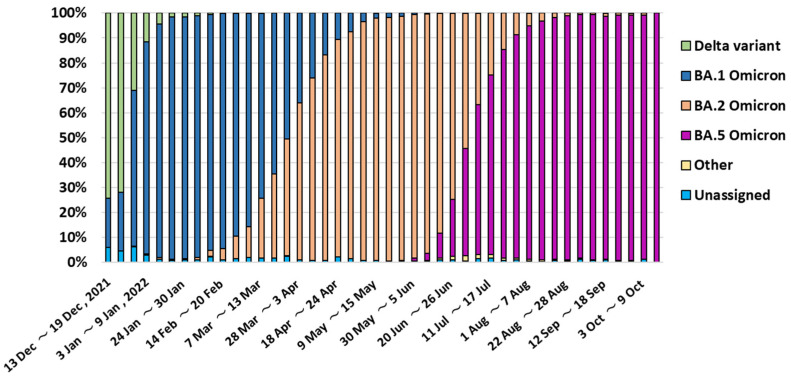
Trends in the changing proportions of the lineage of SARS-CoV-2 in Japan (December 2021~October 2022). These data were obtained from the National Institute of Infectious Diseases [3].

Since the Omicron (B.1.1.529) variant of SARS-CoV-2 was first reported in South Africa on 24 November 2021 [1], it became widely distributed, and by late January, 2022, it was the most prevalent lineage globally, representing 85% of variant cases reported [2]. Japan also experienced a rapid replacement of the Delta variant of concern (VOC) with Omicron, reaching 94% of total newly-detected COVID19 cases by 10–16 January 2022 [3] (Figure A1).

In contrast to its strong infectivity [4,5], a number of epidemiological studies indicated that the clinical severity of infection with the Omicron VOC was lower than with the Delta VOC [6,7,8,9,10,11,12]. However, the clinical severity in the COVID-19 cases was probably influenced not only by the nature of the viral variant but also by booster vaccine coverage [6,13,14,15,16], medical accessibility, availability of novel oral antiviral treatments and life expectancy before the pre-COVID-19 era in the population. The frequency of infection by the BA.1 Omicron sublineage was overtaken by the BA.2 sublineage in April, which was subsequently replaced by BA.5 in late July, being responsible for 97% of the total newly-detected COVID19 cases between 15 and 21 August 2022 in Japan [3] (Figure A1).

These differences in viral sublineage penetration are thought to have altered the pattern of clinical severity of cases with the Omicron variant, thus affecting the case fatality rate (CFR) in the Japanese population. CFR has a very important influence on COVID-19 public health policy decision-making. Davies, M.-A. et al. compared the clinical severity of Omicron BA.4/BA.5 infection with BA.1 in South Africa and concluded that severe hospitalization or risk of death was similar for BA.4/BA.5 and BA.1 infections [17]. However, little is known about the real-world temporal changes of the CFR in cases of infection with different Omicron variants in Japan. Therefore, we aimed to assess the changing case fatality rate of COVID-19 caused by Omicron between January and August 2022 in the Japanese population.

## 2. Materials and Methods

### 2.1. Data Source

**Figure 1:**
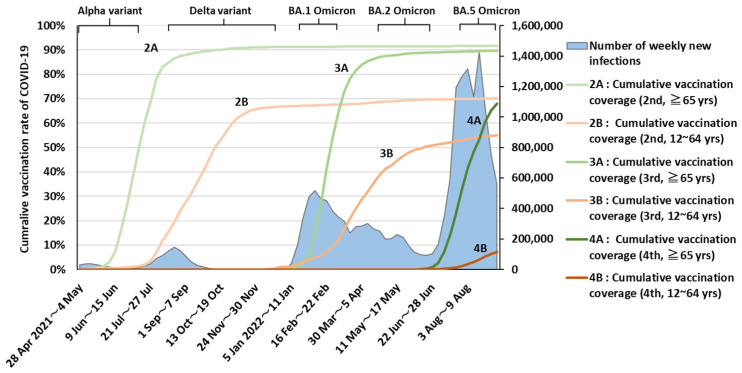
Trends in cumulative vaccination coverage and number of weekly new instances of COVID-19 in Japan. Data on the number of weekly new infections were obtained from the Ministry of Health, Labour and Welfare. Cumulative vaccination rate data were obtained from the Prime Minister’s Office of Japan [20]. Graph indicates dominant period (≧85%).

All instances of test-confirmed COVID-19 were required to be reported by the physician to the Public Health Center (a branch of local government) up until 25 September 2022, adhering to the Infectious Diseases Control Law. Most patients were diagnosed by a test for COVID-19 in an outpatient consultation or at hospital admission. Japanese hospitals test almost all patients for COVID-19 before admission for the purpose of prevention of infection spread in hospitals. Antigen tests were approved on 13 May 2020 and had been already distributed in Japan [18]. Individuals who underwent autopsy were tested. The study team collaborated with 10 prefectures (Gunma, Ibaraki, Nagano, Shiga, Gifu, Osaka, Tokushima, Saga, Nagasaki, and Miyazaki) with a total population of 14.84 million residents. We identified study subjects aged ≥40 years of age with newly test-confirmed COVID-19 between 3 January 2022 (the first date of the first week in 2022) and 28 August 2022 (the last date of the 9th week in 2022). Since the case fatality rate among COVID-19 cases with the Omicron variant aged 39 years and younger was considered to be very low [6], we only included cases for those aged 40 years and over in this trend investigation. Japan experienced a sixth endemic wave that was mainly caused by the BA.1 Omicron sublineage and a seventh endemic wave due to the BA.5 sublineage (Figure 1 and Figure A1).

According to their clinical status, all the SARS-CoV-2-positive symptomatic cases were required to isolate at home, stay in a residence made available by the local government, or in hospital for at least 10 days from the onset and for 72 h after symptom resolution. All test-positive asymptomatic cases were also required to isolate at home for 7 to 10 days. If such asymptomatic cases acquired any symptoms during their isolation period, they were also required to isolate for 72 h after symptom disappearance. Public health nurses in the Japanese public health system monitored the health status of the isolated COVID-19 cases in their jurisdiction mainly using ICT. Death certificates were required to be made out by the physician according to the Infectious Diseases Control Law during the isolation period, regardless of the actual cause of death. This report was sent to the public health center of the jurisdiction in which the deceased had lived. Study subjects were passively followed up until the end of their isolation, date of death, or 28 days after the COVID-19 diagnosis (whichever occurred first) by infectious control departments within the participating public health centers.

The Ibaraki Prefecture Epidemiological Research Joint Ethics Review Committee approved this study.

### 2.2. Calculation of Case Fatality Rate

The case fatality rate (CFR) was calculated using deceased patients as the numerator. We stratified study subjects into 9 subgroups according to the time of COVID-19 diagnosis, beginning from the first 4 weeks (3 to 31 January) through to the 8th 4-week period (18 July to 14 August) and the final 2 weeks (15 to 28 August) in 2022. To characterize the temporal change in the CFR among Japanese COVID-19 patients with the Omicron variant, we calculated age-standardized CFR and its 95% confidence interval [19] using the Japanese population aged ≥40 years in 2022.

To compare age-specific CFR between COVID-19 cases with BA.1, BA.2, or BA.5 sublineage infections, we compared study subjects diagnosed between 3 January and 20 March (a period during which BA.1 was dominant) with those diagnosed 25 April to 19 June (BA.2 dominant) and those diagnosed 18 July to 28 August (BA.5 dominant), representing the subvariant distribution of ≥95% in COVID-19 patients in Japan at those times [3].

### 2.3. Causes of Death

We distinguished COVID-19-related or non-related death or causes unknown. Death related to COVID-19 was defined as having occurred in patients with dyspnea, pneumonia, and hypoxia (<93% oxygen saturation) or a physician’s diagnosis of death from COVID-19. On the other hand, COVID-19 non-related death was determined from the physician’s diagnosis of death from non-COVID-19 disease or sudden death without any pulmonary symptoms. We calculated the proportion of deaths associated with COVID-19 among all the deaths with known causes within in each of the 9 diagnostic time periods.

## 3. Results

**Figure 2:**
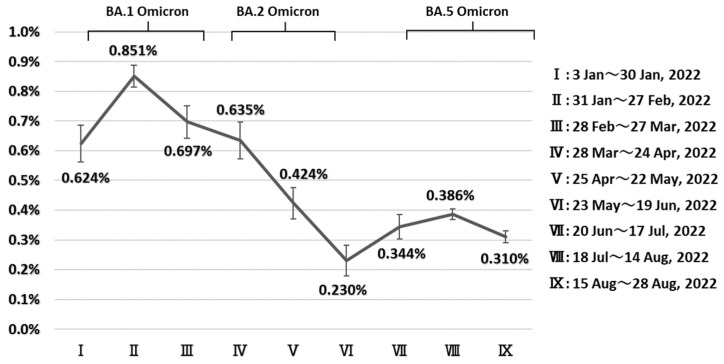
Trends in age-standardized fatality rate in patients aged 40 years infected with the Omicron variant diagnosed between 3 January and 28 August 2022, in Japan. Age-standardization was performed using the whole Japanese population aged 40 years in 2022. Graph indicates dominant period (≧85%).

**Figure 3:**
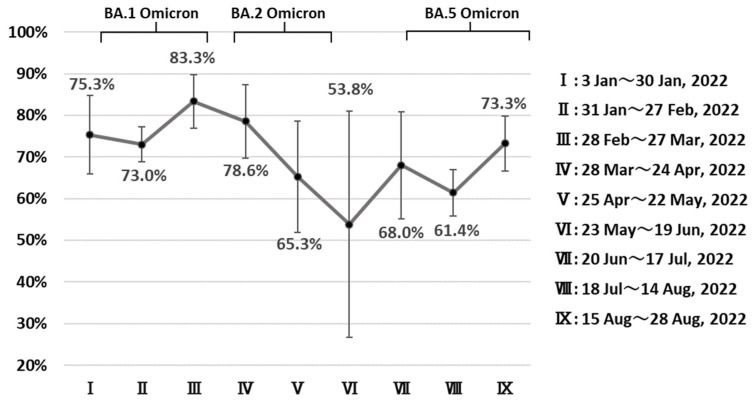
Trend in the proportion of deaths with COVID-19 among deceased individuals aged ≥40 years who were diagnosed with COVID-19 between 3 January and 28 August 2022, in Japan. Graph Indicates dominant period (≧85%).

We extracted data on 552,581 COVID-19 cases diagnosed between 3 January and 28 August 2022 (Table A1). The highest age-standardized CFR (0.85%, 95% confidence interval (CI): 0.78–0.92) was estimated for cases diagnosed in the second 4-week period (31 January to 27 February). The CFR then declined significantly in the 6th 4-week period (0.23%, 95% CI: 0.13–0.33, 23 May to 19 June) (Figure 2). The rate then increased again but remained lower than it was earlier, at 0.39% in the 8th 4-week period (18 July to 28 August). Figure 3 presents trends in the proportion of deaths associated with COVID-19 among all deceased cases with known causes of death. The highest proportion is seen in cases diagnosed in the 3rd 4-week period (83.3%, 95% CI:77.0%–89.7%). This began to decline for cases diagnosed in the 5th period (53.8%, 95% CI:26.7%–80.9%), although this did not reach statistical significance.

**Figure 4:**
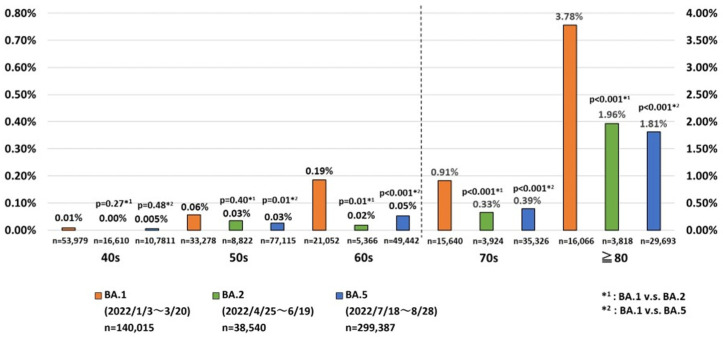
Comparison of fatality rates among Japanese COVID-19 cases infected with one of three sublineages of the Omicron variant in 2022.

The age-specific CFRs for cases with the BA.1 sublineage at ten-year intervals from 40, 50, 60, 70 and ≥80 years were 0.007%, 0.06%, 0.19%, 0.91% and 3.78%, respectively (Figure 4). The CFRs for cases with BA.2 sublineage infections at age 60–70, 70–80 and >80 years (0.02%, 0.33% and 1.96%, respectively) were significantly lower than for BA.1 sublineage infections. Finally, the CFRs in cases infected with BA.5 grouped at ten-year intervals from 50 to ≥80 years of age (0.026%, 0.053%, 0.39% and 1.81%, respectively) were significantly lower than in those with BA.1 sublineage infections in the corresponding age group (Figure 4).

## 4. Discussion

At the introduction of the vaccine, the government decided to give priority to the elderly (≧65 years) (Figure 1) and health care workers. The government of Japan has implemented policies regarding vaccination and antivirals to reduce the fatality rate of COVID-19. BNT162b2 (Pfizer–BioNTech) was approved by the government on 14 February 2021 and was deployed on 17 February 2021 [21,22]. On 21 May 2021, ChAdOx1 nCoV-19 (AstraZeneca) and mRNA-1273 (Moderna) were approved [23]. On 19 April 2022, NVX-CoV2373 (Novavax) was approved [24]. Molnupiravir was approved by the government on 3 December 2021, and the combination of Nirmatrelvir and Ritonavir was approved on 14 January 2022 [25,26]. Our study revealed that the risk of death among COVID-19 cases with the Omicron variant in Japan changed markedly between January and August, 2022. The highest CFR as observed in cases diagnosed from 31 January to 27 February. Japan faced the endemic peak of BA.1 Omicron from 2 to 8 February [27]. Hospital admission rates for COVID-19 patients declined from 14.2% on January 12 to 3.2% on February 9 because of the shortage of in-patient capacity, which possibly affected full accessibility to medical care in COVID-19 patients who actually needed admission [28].

The risk of death started to decrease in March and was reduced to less than one-third in late May to mid-June, relative to earlier in the year. What accounted for this trend? One explanation is likely the timing of increasing SARS-CoV-2 vaccination rates among Japanese aged 65 and over [20] (Figure 1). All Japanese local governments conducted booster COVID-19 vaccination campaigns starting in mid-January, 2022; the cumulative vaccination rate reached 80% by mid-March [20]. Previous COVID-19 vaccine studies showed that protection against infection declines 1 to 2 months after vaccination, whereas protection against severe disease is maintained for much longer [6,13,29]. Therefore, it is plausible that the efficacy of the third COVID-19 vaccination in older Japanese adults in terms of avoiding very severe disease would manifest itself 2 to 3 months after vaccination (i.e., the time when susceptibility to infection is increasing again). The trend for declining proportions of COVID-19-related deaths among all cases seen in essentially the same period may support this notion.

We compared the age-specific risk of death in our cases with BA.1 Omicron cases in a study in England [6] which investigated death within 28 days after confirmed infection between 29 November 2021 and 9 January 2022. This revealed a similar situation in the two studies, at least regarding cases for patients in the 40-to-70 age range [6]. However, the risk of death in people aged 80 and over was significantly lower in Japan than in England (3.78% vs. 5.12%). The lower risk observed in the Japanese may possibly be at least in part attributed to the longer life expectancy before the COVID-19 pandemic in 80-year-old Japanese (10.35 vs. 9.03 year in 2015) [30]. 

The risk of death for cases with BA.5 sublineage infections was significantly lower than for cases with BA.1 at age ≥50 years. This was possibly associated with the increasing COVID-19 vaccination rate (Figure 1), seasonal variation in mortality driven by temperature [31,32], and increasing availability of Molnupiravir and the combination of Nirmatrelvir and Ritonavir this year [25,26] in Japan. Although our descriptive epidemiological approach cannot estimate the impact of the pathogenicity of Omicron sublineage mutants, the low risk of death in patients with BA.5 infections observed in this study may change our view of COVID-19. Countries formulate policies on infection control by considering the impact of COVID-19 on public health and social economic activities. We need to think about the impact on public health in terms of lower current fatality rates.

This study does have some limitations. First, asymptomatic individuals would have been less likely to have been diagnosed than symptomatic patients during the peak endemic period because they would have had less opportunity to be tested under the then-prevailing conditions of insufficient accessibility to testing. This might have induced selection bias that would have increased the apparent risk of death among cases diagnosed in the second and eighth 4-week periods (diagnosed at the peak incidence in the endemic waves) (Figure 1). Second, our data source did not contain individualized information on COVID-19 variants nor Omicron sublineages. Therefore, some misclassification of the sublineage infection was inevitable. However, because we defined sublineage in the study groups based on the time of the dominant penetration of one of the sublineages (≧95%) according to sampling and testing populations from the whole of Japan Figure A1 [3], we believe that the effects of any such misclassification on the risk of death would be minimal. 

Third, as this study design is a descriptive epidemiology that assess the changing Japanese CFR of COVID-19 caused by Omicron, we did not elucidate factors associated with the changing of CFR. Further analytical studies are needed to explore it. Fourth, the accuracy in determining the causes of death was modest because the majority of the COVID-19 cases in this period were isolated in their home or residence provided by the local governments but not in hospital. When the case was isolated in their home or residence, the doctor was less likely to grasp his/her clinical course from the onset. Therefore, the deceased cases who had been isolated at their home or residence possibly had less opportunity to receive a detailed examination, which might have affected the accuracy in determining causes of death. However, as this situation was the same throughout the study period, the influence of this inaccuracy on the proportion of COVID-19-related deaths among the deceased cases would be modest.

## 5. Conclusions

Our analysis shows that the risk of death in patients infected with the Omicron variant declined significantly in Japan in 2022. A very low CFR in cases with BA.5 sublineage infection would change the recognition of this disease as a high-risk pathogen in this country.

## Data Availability

The data presented here is not publicly available due to the privacy policy of the Cooperated Prefectural and Municipal Governments.

## Appendix A

**Table A1:** Age-standardized fatality rate of COVID-19 cases aged ≥40 years caused by the Omicron variant diagnosed between January and August in Japan, 2022.

Diagnosed Period	Number of Cases	Number of Death	Age-Standardized Fatality Rate	95%CI (Lower-Upper)
I (3 January~30 January 2022)	27,674	108	0.624%	(0.563%–0.685%)
II (31 January~27 February 2022)	80,101	557	0.851%	(0.814%–0.888%)
III (28 February~27 March 2022)	39,303	169	0.697%	(0.642%–0.752%)
IV (28 March~24 April 2022)	34,113	110	0.635%	(0.572%–0.697%)
V (25 April~22 May 2022)	24,358	71	0.424%	(0.371%–0.477%)
VI (23 May~19 June 2022)	14,182	21	0.230%	(0.179%–0.281%)
VII (20 June~17 July 2022)	33,463	72	0.344%	(0.302%–0.385%)
VIII (18 July~14 August 2022)	182,366	474	0.386%	(0.368%–0.404%)
IX (15 August~28 August 2022)	117,021	254	0.310%	(0.290%–0.330%)

Age—standardization was performed using Japanese population aged 40 and over in 2022.

**Table A2:** Proportion of deaths with COVID-19 among deceased individuals aged ≥40 years who were diagnosed with COVID-19 between 3 January and 28 August 2022, in Japan.

Diagnosed Period	Cases of COVID-19 Related Deaths	Cases of COVID-19 Non-Related Deaths	Cause of Death Not Known	Total
Ⅰ (3 January~30 January 2022)	61	20	27	108
Ⅱ (31 January~27 February 2022)	314	116	127	557
Ⅲ (28 February~27 March 2022)	110	22	37	169
Ⅳ (28 March~24 April 2022)	66	18	26	110
Ⅴ (25 April~22 May 2022)	32	17	22	71
Ⅵ (23 May~19 June 2022)	7	6	8	21
Ⅶ (20 June~17 July 2022)	34	16	22	72
Ⅷ (18 July~14 August 2022)	183	115	176	474
Ⅸ (15 August~28 August 2022)	126	46	82	254

**Table A3:** Fatality rates of COVID-19 cases caused by one of three sublineages of the Omicron variant in Japan, 2022 (40s, 50s, 60s, 70s and ≧80 years).

	BA.1(B.1.1.529.1) *^1^	BA.2(B.1.1.529.2) *^2^	BA.5(B.1.1.529.5) *^3^	*p*-Value ^(1)^ *^4^	*p*-Value ^(2)^ *^4^
40s	4/53979 (0.00741%)	0/16610 (0.00%)	5/107811 (0.00464%)	0.27	0.48
50s	19/33278 (0.0571%)	3/8822 (0.0340%)	20/77115 (0.0259%)	0.40	0.01
60s	39/21052 (0.185%)	1/5366 (0.0186%)	26/49442 (0.0526%)	0.01	<0.001
70s	143/15640 (0.914%)	13/3924 (0.331%)	139/35326 (0.393%)	<0.001	<0.001
≧80	607/16066 (3.78%)	75/3818 (1.96%)	538/29693 (1.81%)	<0.001	<0.001

*^1^ 3 January~20 March 2022, *^2^ 5 April~19 June 2022, *^3^ 18 July~28 August 2022, *^4^ Equality of proportions hypothesis test ^(1)^ Difference between BA.1 and BA.2 ^(2)^ Difference between BA.1 and BA.5.
